# Role of home blood pressure monitoring in resistant hypertension

**DOI:** 10.1186/s40885-022-00226-1

**Published:** 2023-01-15

**Authors:** Hyue Mee Kim, Jinho Shin

**Affiliations:** 1 Division of Cardiology, Department of Internal Medicine, Chung-Ang University Hospital, Chung-Ang University College of Medicine, Seoul, Republic of Korea; 2 Division of Cardiology, Department of Internal Medicine, Hanyang University Hospital, Hanyang University College of Medicine, 222-1 Wangsimni-ro, Seongdong-gu, 04763 Seoul, Republic of Korea

**Keywords:** Resistant hypertension, Ambulatory blood pressure monitoring, Home blood pressure monitoring, White-coat effect, Self-monitoring, Adherence, Pseudoresistance

## Abstract

The definition of resistant hypertension (RHT) has been updated to include failure to achieve target blood pressure (BP) despite treatment with ≥3 antihypertensive drugs, including diuretics, renin-angiotensin system blockers, and calcium channel blockers, prescribed at the maximum or maximally tolerated doses, or as success in achieving the target blood pressure but requiring ≥4 drugs. RHT is a major clinical problem, as it is associated with higher mortality and morbidity than non-RHT. Therefore, it is crucial to accurately identify RHT patients to effectively manage their disease. Out-of-clinic BP measurement, including home BP monitoring and ambulatory BP monitoring is gaining prominence for the diagnosis and management of RHT. Home BP monitoring is advantageous as it is feasibly repetitive, inexpensive, widely available, and because of its reproducibility over long periods. In addition, home BP monitoring has crucial advantage of allowing safe titration for the maximum or maximally tolerable dose, and for self-monitoring, thereby improving clinical inertia and nonadherence, and allowing true RHT to be more accurately identified.

## Background

Hypertension is associated with cardiovascular events and mortality regardless of socioeconomic status or ethnicity [1, 2]. Owing to various antihypertensive drugs and drug combination strategies, the blood pressure (BP) of many hypertensive patients is well controlled. However, with the aging population and increasing prevalence of chronic kidney disease and obesity, the number of patients with BP that is not within the target range, despite the use of various drugs, is increasing—a condition called resistant hypertension (RHT) [3, 4]. RHT is a major clinical problem as it is associated with poor prognosis, including cardiovascular diseases, end-stage renal disease (ESRD), and hypertension-related target organ damage [5]. Therefore, it is important to identify patients with uncontrolled hypertension by ≥ 3 antihypertensive drugs for an accurate diagnosis and effective management. Although clinical BP measurements are the gold standard approach for the diagnosis and treatment of hypertension, out-of-clinic BP measurement, including ambulatory BP monitoring (ABPM) and home BP monitoring (HBPM), has been practiced for > 30 years, and many guidelines even suggest the application of out-of-clinic BP measurement in the diagnosis and management of hypertension [6, 7]. In general, out-of-clinic BP measurement in RHT, where pseudoresistance needs to be excluded during diagnosis, is gaining prominence [8]. Although many previous studies have recommended ABPM over HBPM [8–10]. HBPM is advantageous for being inexpensive, widely available, and because it allows repeated monitoring to be more feasible over long periods [10]. In particular, both the recent intensification of the target BP and updates in the definition and classification of RHT highlight the role of HBPM [8]. Herein, we review the role of HBPM in RHT diagnosis and management.

## Definition and prevalence of RHT

RHT is defined as uncontrolled BP despite treatment with ≥ 3 antihypertensive agents, including a long-acting calcium channel blocker, renin-angiotensin system blocker, and diuretic, at the maximal or maximally tolerable dosages [6, 8, 11]. This definition also includes BP controlled with ≥ 4 antihypertensive agents as controlled RHT [8]. The reported prevalence of RHT is 8–21%, owing to variations across populations and methodologies [12–15], which make the real prevalence of true RHT difficult to determine. In contrast, the reported prevalence of RHT among those who adhered to lifestyle modifications to control their BP was only 0.8% in the MINISAL-SILA study [16]. Pseudoresistance, which cannot be completely ruled out, may have a critical influence in this broad prevalence. Despite known limitations in the ability to accurately discriminate pseudoresistance, according to a previous study, ≤ 50% of patients with RHT may not have true RHT [17]. Thus, the RHT guidelines highlight the white-coat effect and drug nonadherence as representative causes of pseudo-RHT that should not be included when diagnosing RHT [7, 8]. To determine the actual prevalence of RHT, additional epidemiological studies are warranted, including the assessment of pseudoresistance and consideration of lifestyle.

## Pseudoresistance

Many uncontrolled hypertension cases are not true RHT cases [17]. Falsely elevated BP levels despite the use of ≥ 3 antihypertensive agents may appear as uncontrolled hypertension—this is known as pseudo-RHT. The most common causes of pseudo-RHT are an inaccurate BP measurement technique, drug nonadherence, being under treatment, and white-coat effects [18]. Errors in BP measurement are common in routine clinical practice. Environmental settings, use of an incorrectly sized cuff, and technical issues, for example deflating the BP cuff too fast, often result in falsely elevated BP. The white-coat effect is defined as an office BP above target but an out-of-clinic BP below target. A previous study reported that approximately 40% of patients with apparent RHT had white-coat RHT, as found with ABPM [15]. Therefore, out-of-clinic BP and self-monitored BP are required to rule out pseudo-RHT. Nonadherence must also be excluded before diagnosing RHT. Jung et al. [19] estimated that 50% of cases with apparent RHT were drug nonadherence cases, a finding supported by other studies [20, 21]. Additionally, nonadherence is directly related to the number of tablets prescribed [22]; thus, the nonadherence rate may increase under uncontrolled hypertension, where the number of drugs prescribed increases.

## Prognosis and clinical importance of accurate RHT diagnosis

The prognosis of patients with RHT is worse than that of patients with general hypertension [5]. However, it is not yet clear whether this poor prognosis is simply owing to high BP or to other pathophysiologies or comorbidities. RHT patients also have a high rate of comorbidities such as diabetes, chronic kidney disease, ischemic heart disease, and cerebrovascular disease, which are all known risk factors for cardiovascular diseases [5, 23]. Additionally, the number of prescribed antihypertensive medications can increase cardiovascular outcomes regardless of BP level [24]. Thus, not only RHT itself but also these characteristics may affect the poor outcomes. In an RHT cohort including 60,327 subjects, RHT increased the risk of ischemic heart disease, congestive heart failure (HF), cerebrovascular accident, ESRD, and all-cause mortality when compared with general hypertension [5]. Similarly, Yoon et al. [25] showed that refractory hypertension, defined as uncontrolled BP despite the use of ≥ 5 antihypertensive medications, and resistant hypertension were associated with a higher risk of cardiovascular and all-cause mortality than nonresistant hypertension. Another long-term (10 years) follow-up study of RHT also showed that RHT was associated with a high rate of major adverse events [26]. In addition, Kario et al. [27] reported a significantly higher risk for HF in true RHT patients diagnosed with ambulatory BP monitoring without a history of HF. Interestingly, controlled RHT showed favorable outcomes when compared with uncontrolled RHT [5]. Patients with uncontrolled RHT had a greater risk of cerebrovascular accident and ESRD than those with controlled RHT, although the risks of ischemic heart disease, congestive HF, and all-cause mortality were similar [5]. In accordance with these findings, Cardoso et al. [28] evaluated HBPM as a predictor of cardiovascular outcomes in patients with RHT and demonstrated that higher or uncontrolled home BP levels are associated with adverse outcomes. Similarly, Tsioufis et al. [29] demonstrated that persistent RHT is independently associated with adverse cardiovascular prognosis, while resolved and incident RHT are not. Taken together, these studies provide evidence that the presence of RHT is an important predictor of cardiovascular diseases in patients with hypertension. In addition, studies regarding controlled or resolved RHT suggest that the prompt resolution of RHT is needed to improve clinical outcomes [5, 28, 29].

## Diagnostic approach of resistant hypertension

An accurate RHT diagnosis is crucial, considering its prognosis and the need for additional diagnostic tests and treatment improvement. Recognizing pseudo-RHT is also important for patients whose BP is falsely elevated. If pseudo-RHT is not properly excluded, additional diagnostic tests and antihypertensive medications could be prescribed to patients who do not have true RHT, potentially increasing the risk of adverse events and creating unnecessary costs [30].

**Fig. 1:**
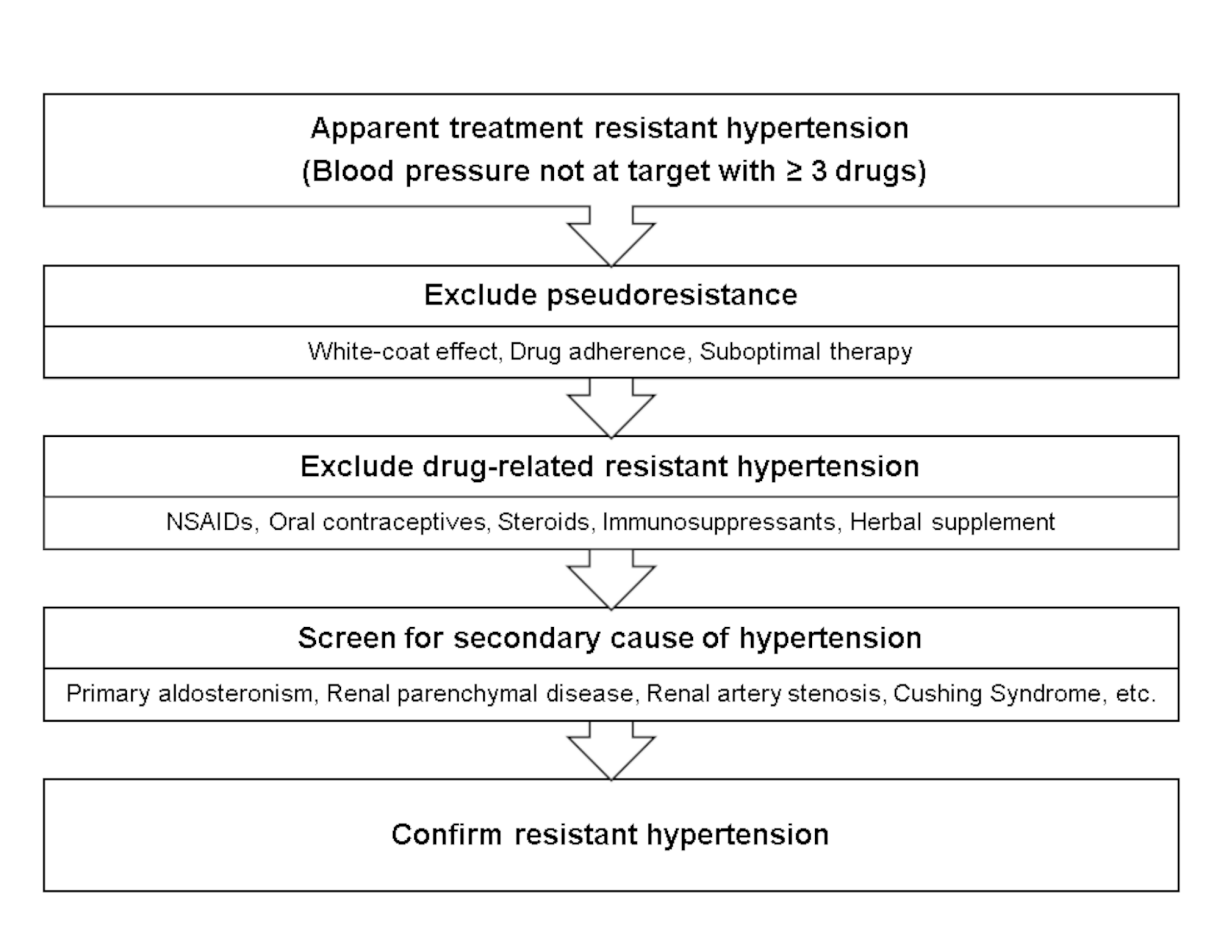
Diagnostic algorithm for a patient with suspected resistant hypertension. NSAID, nonsteroidal anti-inflammatory drug

When evaluating patients with RHT, physicians should consider several steps before confirmation of true RHT (Fig. 1) [8, 31]. Excluding pseudoresistance is the first step in the diagnosis of RHT, and ABPM and HBPM are recommended, as they can rule out white-coat effects. Nonadherence to antihypertensive medication should be assessed through patient interviews. After pseudoresistance is excluded, drug-related RHT should be assessed. Several medications can influence high BP and lead to RHT. Nonsteroidal anti-inflammatory drugs, steroids, immunosuppressive agents, oral contraceptives, and herbal supplements are representative examples of drugs that can aggravate RHT. Additionally, primary aldosteronism, renal parenchymal disease, renal artery disease, Cushing syndrome, and obstructive sleep apnea should be considered as possible secondary causes of RHT. A medical interview and physical examination are essential to identify secondary causes of hypertension.

## Characteristics of out-of-office BP measurements for diagnosis of RHT

The most important and difficult step in RHT diagnosis is identifying white-coat effects and drug nonadherence, which are the most representative pseudoresistance cases. Out-of-clinic BP measurement, including ABPM and HBPM, can play a key role in this step. The characteristics of these two methods are listed in Table 1. Both ABPM and HBPM show higher measurement frequency than clinic BP and are typically used to assess average BP outside the clinic and without a health care provider, allowing discrimination of discrepancies between clinic and out-of-clinic BP measurement. ABPM can identify nighttime hypertension and BP during the patient’s daily routine. However, some patients do not tolerate this, owing to life and sleep disturbances. Moreover, the necessary equipment is not widely available and/or expensive in some countries. Additionally, ABPM requires > 2 clinic visits for set up and return of the device. HBPM can be measured under standard conditions, achieving stable mean values and high reproducibility. Therefore, HBPM can provide feedback on the diagnosis and help in hypertension control. However, some home devices are not validated and require patient training [10, 32] Evidence of the HBPM in clinical practice has been documented in many studies, and HBPM will become more useful and widely used if the measurement techniques are well-known to patients. Table 2 provides recommendations for HBPM.

**Table 1:** Characteristics of ambulatory and home BP monitoring

Characteristic	Clinic BP	Ambulatory BP	Home BP
No. of measurements	Low	High	High
Reliability	Low	Medium	High
Repeatability	High	Low	High
Standardization	Low	High	Low
Device validation	High	High	Variable
Reflection of prognosis	Weak	Adequate	Adequate
White-coat effect	Not available	Available	Available
Masked hypertension	Not available	Available	Available
Nocturnal hypertension	Not available	Available	Possibly available
Morning surge	Not available	Available	Possibly available
Self-monitoring	No	No	Yes
Drug adherence	No effect	No effect	Improving
Telehealth	Not available	Not available	Available

BP, blood pressure

**Table 2:** Recommendations for home blood pressure monitoring

Procedure	Recommendation
Precaution	No tobacco or caffeine consumption, or heavy exercise 30 min before measurement After voiding Use of an upper arm cuff Place the arm at heart level, with supported back and feet flat on the ground After 2–5 min of rest
Time	Morning: within 1 h after waking up, before taking antihypertensive drugs, and before breakfast Night: before bedtime Before taking a shower/bath
Schedule and frequency	7-Day measurements, 2–3 times per session Diagnosis: ≥1 wk Follow-up treatment: 5–7 day preceding the clinic visit

## HBPM use for RHT diagnosis

Traditionally, ABPM is considered the first-line diagnostic tool and gold standard method for determining white-coat effects among out-of-clinic BP measuring methods [8–10], as it can distinguish true hypertension from white-coat hypertension, both in treated and untreated patients [33]. Cardiovascular events are found less often in patients with white-coat hypertension than in those with elevated ambulatory BP [34, 35]. HBPM also shows good agreement with ABPM in RHT diagnosis [36–38], and is recommended as a complementary method in several guidelines. Nonetheless, HBPM can provide timely and clinically relevant data, while having higher reproducibility than ABPM [32]. Thus, in patients with apparent RHT, HBPM may be more useful than ABPM for excluding white-coat effects. HBPM can also be used in the evaluation of drug adherence, another important factor. Various direct and indirect methods for evaluating nonadherence, such as direct measurement of drugs or biological markers, counting pills, or electronic monitoring systems, have been developed, but these are not accessible to all patients. A previous study suggested that differences between out-of-clinic and clinic BP help to identify drug nonadherence in patients with apparent RHT [39]. A large difference between clinic and home BP can indicate poor antihypertensive drug adherence in patients with uncontrolled hypertension. Although in the past only RHT identified by ABPM was proven to be associated with cardiovascular risk, recently, Narita et al. [40] demonstrated that there is an association between RHT as identified on HBPM and cardiovascular events. Similarly, sustained apparent RHT and masked uncontrolled RHT detected on HBPM are associated with cardiovascular morbidity and mortality [41]. Thus, HBPM might be useful for diagnosis and risk stratification of patients with RHT.

Despite this evidence, Wei et al. [9] discussed why HBPM cannot replace ABPM for RHT diagnosis. First, they reported that HBPM does not enable easy BP recording during the night, which is critical to avoid adverse cardiovascular outcomes. However, nocturnal BP measurement using ABPM showed poor reproducibility owing to the influence of the quality and quantity of nocturnal sleep [42, 43]. Moreover, the morning surge caused by differences between morning and nocturnal BP was not reproducible either [44]. Technological developments in home BP devices allow the measurement of nocturnal BP. Although not yet widely commercialized, preliminary evidence shows that nocturnal HBPM is feasible and has an ability to detect non-dippers, similar to ABPM [45]. In addition, Narita et al. [46] recently demonstrated that nighttime BP measured by the home BP device was associated with cardiovascular risk in patients with true RHT. In particular, nocturnal hypertension is known to be an important factor in RHT patients with sleep apnea [47], and it is expected whether HBPM can be used to monitor it in the future. Next, the authors of the study argued that the diagnosis of isolated nocturnal hypertension can be estimated by only ABPM. However, as mentioned above, if nocturnal HBPM becomes more widely feasible, then ABPM would not be the only way to detect isolated hypertension. Finally, they insisted that HBPM could miss diagnosing a masked or sustained hypertension. However, in 1996, Ohasama detected masked hypertension with HBPM in the general population. Masked hypertension detected with HBPM predicted target organ damage and the future development of sustained hypertension [48, 49]. Additionally, the poor prognosis of cardiovascular events in patients with masked hypertension distinguished by HBPM was also confirmed in the apparent RHT population [41]. Collectively, HBPM may not only play a supporting role in the diagnosis of RHT but could also be used as a viable alternative, similar to ABPM.

## Role of HBPM in overcoming therapeutic inertia in RHT

The current definition of RHT in major guidelines, which mandate the use of the optimal doses of three drugs [6, 11], is inherently limited by the obscure definition of “optimal.” If the physician defines the dose as optimal, the case will be diagnosed as apparent RHT, regardless of the actual dose. Even if pseudo-RHT is excluded by using HBPM and nonadherence is resolved, the prevalence of RHT could vary widely depending on the level of up-titration to the maximal dose [50]. As most guidelines recommend combinations of multiple lower dosages rather than titration to the maximal dose of one drug, followed by the stepwise addition of another drug, physicians may not be used to up-titrating the three drugs to the maximum or maximally tolerated doses in real clinical practice. Thus, therapeutic inertia or failure to up-titrate to the maximally tolerated doses might also contribute to inaccurate diagnosis of apparent RHT. Concern around the safety of maximal doses also could be the reason for clinician’s hesitance or clinical inertia when the titration depends only on clinic BP. Moreover, as the target BP has recently become 130/80 mmHg or lower for an increasing number of patients, the influence of therapeutic inertia on the diagnosis of apparent RHT is likely to increase.

In practice settings wherein HBPM is not available, therapeutic inertia may originate from the knowledge that the white-coat effect is one of the major causes of apparent RHT. HBPM is a useful tool for overcoming therapeutic inertia during the diagnosis of true RHT. HBPM is more feasible than ABPM for repeated monitoring, as it allows the titration of antihypertensive medication until the target BP has been achieved, by excluding overtreatment for white-coat uncontrolled hypertension and avoiding therapeutic inertia with regard to sustained uncontrolled hypertension or masked uncontrolled hypertension. Poor clinical tolerability when up-titrating drugs up to the maximal dose could imply the possibility of white-coat effects. Thus, considering the potential hazard of hypotension in white-coat uncontrolled hypertension when increasing the dose according to clinic BP, it would be safer to use HBPM routinely before up-titration to the maximal dose than to pose risks from hypotensive side effects. However, whether masked uncontrolled hypertension categorized by HBPM despite the administration of the three drugs at maximal doses could be regarded as RHT has not yet been clearly defined by the major guidelines.

## HBPM for RHT management

Although HBPM is considered to have major value in diagnosis; there is abundant evidence that it is useful for improving BP management in hypertensive patients. In particular, it can play an important role in RHT cases where BP is not well controlled. First, HBPM may provide information on the response to antihypertensive medications [32, 51]. HBPM is recommended to be measured twice, in the early morning and evening. This way it is possible to evaluate whether BP is well controlled during the whole day. Second, HBPM is associated with better hypertension control. Previous studies and meta-analysis showed that HBPM decrease systolic BP significantly more than ordinary treatment and promoted achievement of target BP [52–55]. This may be explained by the fact that measuring home BP may improve drug adherence and that a patient’s HBPM recording may motivate physicians to provide a more active treatment. Marquez-Contreras et al. [56] reported that HBPM can improve drug adherence in patients with hypertension. Similarly, Zhang et al. [57] also demonstrated HBPM improved treatment adherence and BP control despite similar antihypertensive treatment. Recently, the logic and the behavioral mechanism behind the influence of HBPM on adherence has begun to be investigated [58]. Appropriate feedback for HBPM reading may increase the patient’s perception of the efficacy of antihypertensive drugs on BP level, thereby improving adherence. Third, HBPM reduced medication use leading to lower medication costs [59]. HBPM leads to lower prescription of antihypertensive medication without increasing office BP and target organ damage. Since most antihypertensive drugs are supposed to be taken life long, the reduced medication cost associated with HBPM is expected to be significant. Fourth, with increasing use and development of digital health technologies, HBPM telemonitoring and smartphone applications with HBPM may offer additional benefits [6, 11]. A meta-analysis on different telehealth interventions revealed that the effects of telehealth on BP lowering were significantly greater than those of HBPM without intervention [60, 61]. Although digital health has not been established as a standard protocol yet, in the future, use of digital health in the form of HBPM will likely become increasingly involved in controlling BP.

## Conclusion

The clinical importance of out-of-clinic BP in RHT cannot be overemphasized. Until recently, ABPM was considered better than HBPM for the diagnosis of hypertension. However, HBPM has various strengths when measuring out-of-clinic BP. HBPM can be easily repeated and used over longer periods to assess variability, is cheap, and widely available. The repeatability of HBPM can promote safe clinical practice during active up-titration to the maximal dose to reach the target BP, and self-monitoring by HBPM can increase drug adherence. Furthermore, continuous technological developments allow measuring night BP at home. In the future, telehealth advances such as telemonitoring, smartphone applications, and smart watches will allow wider use of HBPM. These characteristics will make HBPM an optimal method for diagnosis and management of hypertension, especially RHT.

## Data Availability

Not applicable.

## Abbreviations

ABPM: Ambulatory blood pressure monitoring
BP: Blood pressure
ESRD: End-stage renal disease
HBPM: Home blood pressure monitoring
RHT: Resistant hypertension
